# A mechanistic evaluation of the traditional uses of *Nepeta ruderalis* in gastrointestinal and airway disorders

**DOI:** 10.1080/13880209.2017.1285325

**Published:** 2017-02-09

**Authors:** Hassan Mahmood, Mueen Ahmad Chaudhry, Zeeshan Masood, Muhammad Asad Saeed, Sherjeel Adnan

**Affiliations:** a Faculty of Pharmacy, The University of Lahore, Lahore, Pakistan;; b Faculty of Pharmacy, University of Sargodha, Sargodha, Pakistan

**Keywords:** Spasmolytic, broncho-relaxant, antidiarrhoeal, calcium channel-blocking activity

## Abstract

**Context:***Nepeta ruderalis* Buch.-Ham. (Lamiaceae), locally known as Badranj Boya, is an aromatic herb used traditionally as an antispasmodic, antidiarrhoeal, and anti-asthamatic remedy.

**Objective:** Aqueous methanolic extract of *N. ruderalis* was studied to investigate its traditional uses.

**Materials and methods:** Study was conducted from September 2015 to February 2016. *In vitro* spasmolytic and broncho-relaxant activity of crude extract of *N. ruderalis* (whole plant) was evaluated at 0.01–10 mg/mL final bath concentration in isolated rabbit jejunum and tracheal tissues, using PowerLab data acquisition system (Transonic Systems Inc., Ithaca, NY). *In vivo* antidiarrhoeal activity was evaluated in castor oil-induced diarrhoeal mice at the dose of 300 and 500 mg of crude extract orally.

**Results:** Crude extract of *N. ruderalis* completely relaxed spontaneously contracting, high K^+^ (80 mM) and carbachol (1 μM) induced contracted jejunum with an EC_50_ value of 5.85 (5.45–6.27), 4.0 (3.80–4.23) and 2.86 (2.48–3.29), similar to verapamil. Nr.Cr relaxed high K^+^ and carbachol induced contractions, at 5 and 10 mg/mL with an EC_50_ value of 2.37 (2.11–2.67) and 3.26 (2.9–3.67), respectively, and also displaced calcium concentration–response curves toward right at 0.1 and 0.3 mg/mL. Nr.Cr exhibited antidiarrhoeal protection at a dose of 300 and 500 mg/kg, similar to verapamil, whereas no acute toxicity signs were seen up to 5 g/kg in healthy mice.

**Discussion and conclusion:** Results suggest the presence of spasmolytic and broncho-relaxant effects in the crude extract of *N. ruderalis*, possibly mediated through calcium channel-blocking activity, providing the pharmacological basis for its traditional uses in gastrointestinal and airway disorders.

## Introduction

Conventional drugs, used commonly in the treatment of many disorders, are not always safe or effective, and sometime beyond the affordability or access of large no of people in the world (Rehman et al. 2012). Traditional systems of medicine, mainly based on the use of herbs as medicine, are one of the most popular alternative options for such patients. There is a need to evaluate new, economical, and cost-effective drugs to meet the future challenges regarding diseases, despite the availability of several remedies obtained from synthetic sources. According to the World Health Organization (WHO), the use of herbal medicines in the health care system is increasing with the passage of time (Mehmood et al. 2011a). It is sound to believe that herbal medicines are relatively safe as compared with chemical substances because these are known to contain side effects neutralizing potential (Mehmood et al. 2011b). Scientific validations of traditional herbal products are also attracting the prescribers of modern medicines towards the use of neutraceuticles in their practices, globally (Rehman et al. 2012).

*Nepeta ruderalis* Buch.-Ham. ex Benth. (Lamiaceae), syn. *Nepeta hindostana* Haines, locally known as Badranj boya (Khare 2007), is an aromatic herb with bitter taste used traditionally in many Ayurvedic and Yunani medicines. *Nepeta ruderalis* occurs in temperate parts of western Afghanistan and tropical, it is found in India, Pakistan, Nepal, and west Himalayas (Kew 2014). The plant is known for uses such as cardiac, brain, and gastric tonic. It is a blood purifier and relieves high blood pressure. It has anti-asthmatic, anti-catarrhal, and sedative properties (Awan 1960). It is used to treat fever, body ache, diarrheoa, dysentery, as a carminative and antispasmodic agent (Quattrocchi 2012), as a gargle for sore throat and bad breath, also to treat gonorrhoea (Nadkarni 1996). The plant also has hypocholesterolaemic and central nervous system (CNS) depressant effects (Khare 2007).

Some scientific studies have already proven the medicinal importance of *N. ruderalis* as an antimicrobial, anti-oxidant, and antipyretic (Koche 2010; Pandey et al. 2015). Phytochemical studies revealed the isolation of nepehinol (Ahmad et al. 1985) and, nepehinal, a triterpenoidal aldehyde from the plant (Ahmad et al. 1993). The present study was undertaken to validate traditional uses of *N. ruderalis* in the gastrointestinal tract (GIT) and respiratory disorders.

## Materials and methods

### Drugs and chemicals

All chemicals of research grade were used for experimental work. Magnesium chloride, potassium dihydrogen phosphate, sodium bicarbonate, potassium chloride, magnesium sulphate, glucose, sodium dihydrogen phosphate (Merck, Darmstadt, Germany), and sodium chloride (BDH Laboratory Supplies, Poole, England) were used for physiological solution preparation. Carbachol (CCh), acetylcholine chloride (Ach) and loperamide (Sigma Chemical Co., St. Louis, MO) were utilized as standards and castor oil (Marhaba Laboratories, Lahore, Pakistan) for the induction of diarrhoea. Whereas chloroform, hydrochloric acid, benzene, sulphuric acid, iodine, picric acid (BDH Laboratory Supplies, Poole, England), ammonium hydroxide, ferric chloride (Sigma Chemical Co., St. Louis, MO), ethanol, ammonia solution (Saba Saleem Industries and Fine Chemicals, Karachi, Pakistan), sodium hydroxide (Sigma Aldrich Laborchemikalien GmbH, Steinheim am Albuch, Germany), benzene, lead acetate, and potassium iodide (Merck, Darmstadt, Germany) were used in phytochemical analysis of crude extract.

Distilled water was used for the preparation of standard solutions, dilution, and physiological salt solutions (Tyrode’s and Kreb’s solutions). Physiological salt solutions and dilutions of standard drugs were freshly made on the day of the experiment by the method of Naz et al. (2016).

### Plant material and crude extract preparation

Dried *N. ruderalis* whole plants were procured in April 2015 from a reputed herb store in Lahore, Pakistan. Plant material was identified by taxonomy expert Prof. Dr. Zaheer-ud-din Khan, Department of Botany, GC University, Lahore. Specimen voucher (Fl. P. 644-4) was submitted in Faculty of Pharmacy, The University of Lahore, Lahore, Pakistan. About 900 g coarse powder of the plant was soaked in approximately 2500 mL, 70% aqueous methanolic solution for 3 d with daily mixing and then filtered. This filtrate was concentrated to thick semi-solid mass by using a rotary evaporator as previously followed by Williamson et al. (1998). The yield of crude extract was 7.78% approximately.

### Animals

Locally bred rabbits (1.0–1.5 kg) and albino mice (25–30 g) were used for *in vitro* and *in vivo* studies were obtained from the animal house of Faculty of Pharmacy, The University of Lahore, Lahore, Pakistan, under controlled environmental conditions. All experiments were performed according to the guidelines of The Institute of Laboratory Animal Resources, National Research Council (Clark et al. 1996), after getting approval (IAEC-2014-01) from the Institutional Animal Ethics Committee of The University of Lahore, Lahore, Pakistan.

### *In vitro* experiments

Rabbits were killed humanely after 24 h of fasting and tissues (jejunum or trachea) were isolated and a portion of tissue was cut and hooked in a tissue, organ bath, pre-filled with 20 mL of physiological solution, continuously aerated with (95% O_2_ and 5% CO_2_) carbogen, sustained at a temperature of 37 °C. 1 g preload was given to the tissue and equilibrated for approximately 30–40 min before giving any further treatment. Tissue was then stabilized by administering the sub-maximal concentration of (0.3 μM) acetylcholine or charbachol (1 μM) 3–5 times and then standard protocols were applied to study the effects of crude extract and verapamil on the isolated jejunum and trachea in triplicate.

### Determination of calcium blocking activity

High K^+^ (80 mM) was used to determine, either calcium channel-blocking mechanism is involved in the relaxant effect of the crude extract. Once the sustained contraction was achieved after addition of high K^+^ in the tissue, organ bath, the crude extract was added in a cumulative fashion. An agent that inhibits the contractions produced by high K^+^ is considered to be a calcium channel blocker (CCB) (Bolton 1979). To confirm the presence of Ca^+2^ channel blocking activity of the crude extract, calcium concentration–response curves (CRCs) were developed in the absence and presence of crude extract as explained previously by following Van Rossum (1963). The tissue was stabilized in Tyrode’s solution, which was replaced with Ca^+2^-free Tyrode’s solution (Tn), to make the tissue Ca^+2^ free. Following an incubation period of 30 min, after replacing Tn with Ca^+2^ free but K^+^ rich Tyrode’s solution (Tr), CRCs of Ca^+2^ were constructed by adding CaCl_2_ in cumulative fashion in tissue organ bath. When two consecutive superimposed control Ca^+2^ curves were constructed, the tissue was incubated in the crude extract for 50 min to evaluate the possible CCB activity. Ca^+2^ CRCs were reconstructed in the presence of different concentrations of Nr.Cr and any right word shift in the curve or suppression in maximum contractile response will be considered as an indicator for the presence of CCB activity.

### *In vivo* experiments

All *in vivo* experiments were performed on overnight fasted albino mice by following the procedures described previously by Mehmood et al. (2011a).

### Acute toxicity test

Mice (*n* = 20) were segregated into four groups. Group one was given normal saline 10 mL/kg (body weight) orally (negative control). Three other groups were given increasing doses of crude extract of *N. ruderalis* (1, 3, and 5 g/kg, equivalent to 12.85, 38.57, and 64.25 g of dried plant) and given free access to food and water. Animals were observed for toxicity signs consistently for 6 h, whereas mortality was noted up to 24 h (Khan & Gilani 2009).

### Antidiarrhoeal activity

Overnight fasted mice (*n* = 25) were segregated into five groups and housed individually in separate cages. The first group was given normal saline (10 mL/kg) orally (negative control). Test groups were administered 300 and 500 mg/kg of crude extract equivalent to 3.85 and 6.42 g of dried plant, respectively. While positive control groups were administered verapamil (50 mg/kg) and loperamide (10 mg/kg). After 1 h, each animal was then given (10 mL/kg, p.o.) castor oil to induce diarrhoea and monitored for defecation up to 6 h (Janbaz et al. 2013). Defecations were counted in each mouse cage and antidiarrhoeal effect was calculated on the basis of diarrheal scoring as following; score 0: no diarrhoea, score 1: soft but formed faeces, score 2: very soft stool, score 3: very loose faeces, and percentage protection against castor oil induced diarrhoea (Naz et al. 2016).

### Statistical analysis

All experimental data were stated as mean ± SEM, whereas median effective concentrations (EC_50_ values) and one-way ANOVA) with 95% confidence intervals (CI) were used to determine difference in various doses followed by Dunnett’s test, *p* value <0.05 was considered statistically significant.

## Results

### *In vitro* experiments

#### Effect on rabbit jejunum

**Figure 1.**
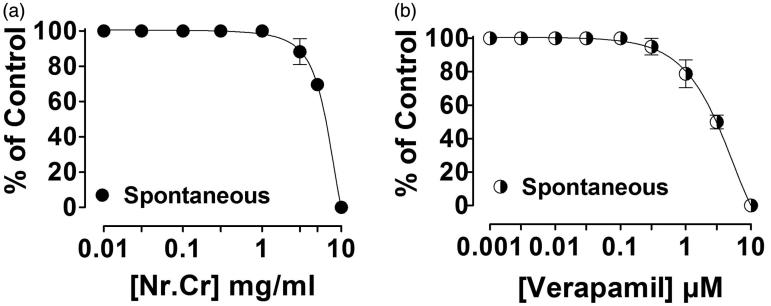
Concentration-dependent inhibitory effect of (a) crude extract of *N. ruderalis* (Nr.Cr) and (b) verapamil, on spontaneously contracting isolated jejunum. Values are expressed as mean ± SEM, *n* = 4–5.

**Figure 2.**
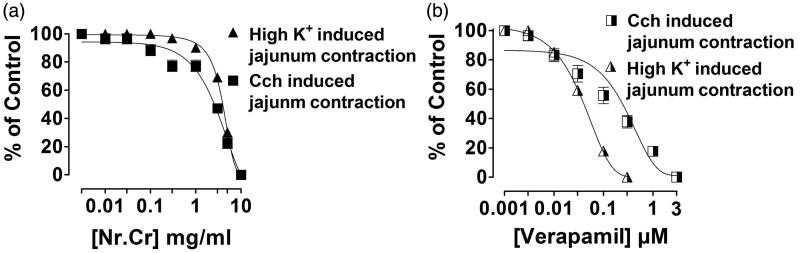
Concentration-dependent inhibitory effect of (a) crude extract of *N. ruderalis* (Nr.Cr) and (b) verapamil on high K^+^ (80 mM) and carbachol (1 μM) induced pre-contracted isolated jejunum. Values are expressed as mean ± SEM, *n* = 4.

Spasmolytic effect of *N. ruderalis* crude extract was observed in isolated rabbit jejunum, when given in cumulative concentration (0.01–10 mg/mL) (Figure 1(a)), similar to verapamil (0.003–10 μM) (Figure 1(b)) with an EC_50_ value of 5.85 (95% CI, 5.45–6.27, *n* = 5) and 3.04 (2.45–3.76, *n* = 4). Whereas high K^+^ (80 mM) or carbachol (1 μM)-mediated contraction in isolated rabbit jejunum was completely relaxed with an ascending concentration of *N. ruderalis* crude extract at 10 mg/mL, with EC_50_ values of 4.0 (3.80–4.23, *n* = 4) and 2.86 (2.48–3.29, *n* = 4) (Figure 2(a)), similar to verapamil at 0.003–0.3 μM and 0.003–3 μM with an EC_50_ value of 0.039 (0.034–0.045, *n* = 4) and 0.25 (0.22–0.29, *n* = 4), respectively (Figure 2(b)).

#### Effect on rabbit trachea

**Figure 3.**
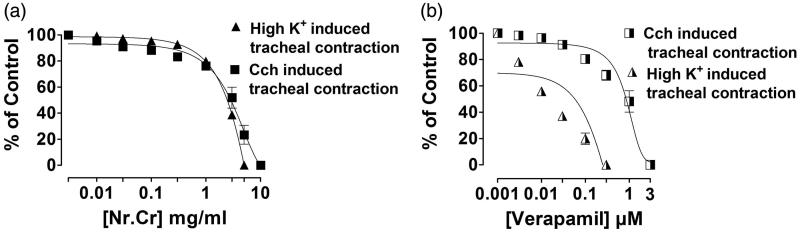
Concentration-dependent inhibitory effect of (a) crude extract of *N. ruderalis* (Nr.Cr) and (b) verapamil on high K^+^ (80 mM) and carbachol (1 μM) induced pre-contracted isolated trachea. Values are expressed as mean ± SEM, *n* = 5.

**Figure 4.**
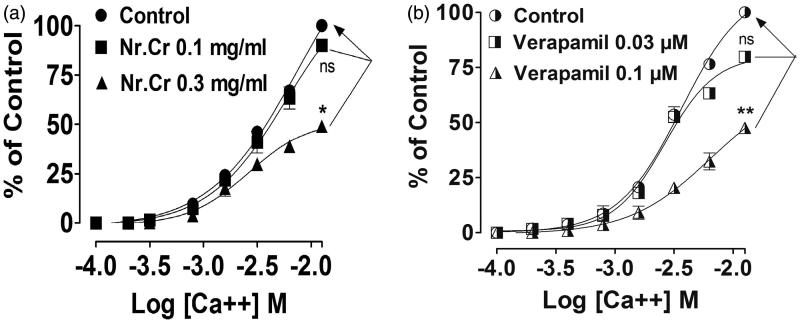
Concentration–response curves of Ca^+2^ (CRCs) in the absence and the presence of (a) crude extract of *N. ruderalis* (Nr.Cr) and (b) verapamil on isolated rabbit trachea. Values are expressed as mean ± SEM, *n* = 3, *p < 0.05, **p < 0.01 versus control, one-way ANOVA, followed by Dunnett’s test.

*Nepeta ruderalis* crude extract showed concentration-dependent relaxation of high K^+^ (80 mM) or carbachol (1 μM)-mediated contractions, when treated on the isolated rabbit trachea. Tissue was fully relaxed at the dose of 5 and 10 mg/mL with an EC_50_ value of 2.37 (2.11–2.67, *n* = 5) and 3.26 (2.9–3.67, *n* = 5) (Figure 3(a)), similar to verapamil (0.003–0.3 μM) and (0.003–3 μM) with an EC_50_ value of 0.058 (0.046–0.067, *n* = 3) and 0.79 (0.60–1.05, *n* = 5) (Figure 3(b)), respectively. A rightward displacement in calcium CRC was shown in the presence of *N. ruderalis* crude extract at 0.1 mg/mL, followed by a significant (*p* < 0.05) suppression in CRC maximum response at 0.3 mg/mL (Figure 4(a)), similarly in the case of verapamil, where the rightward shift was seen at 0.03 μM, followed by a significant (*p* < 0.01) suppression in CRC maximum response at 0.1 μM (Figure 4(b)).

### *In vivo* experiments

#### Acute toxicity test

The crude plant extract of *N. ruderalis* was well tolerated by the test animals up to the tested dose of 5 g/kg orally. No signs of acute toxicity like any behavioral changes, restlessness, seizures, or blindness were present over the period of 4–6 h of observation. Moreover, no mortality was recorded up to 24 h.

#### Antidiarrhoeal activity

**Figure 5.**
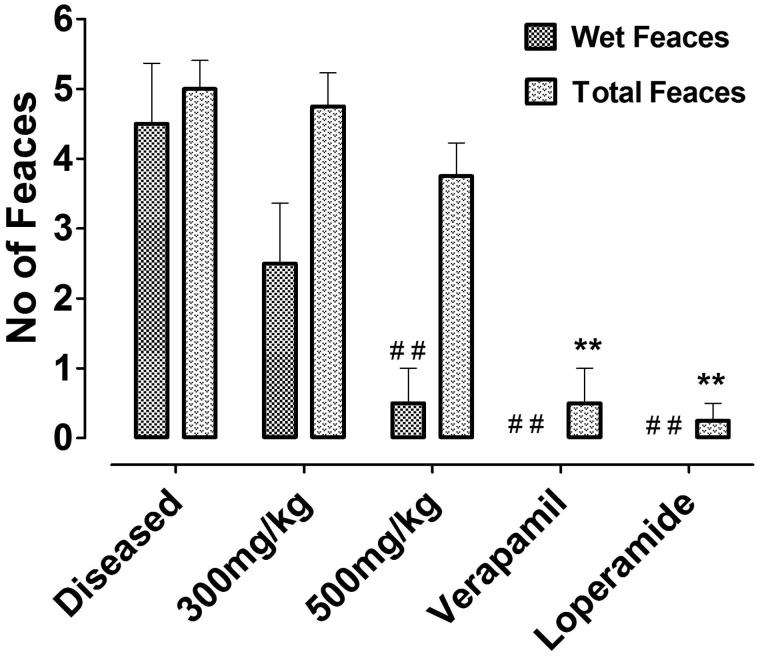
Counted number of wet and total faeces of each test group, exhibiting the results from left to right of negative control, 300 mg/kg, 500 mg/kg of crude extract of Nr.Cr, verapamil, and loperamide. Values are expressed as mean ± SEM, ##*p* < 0.01 versus wet faeces and **p < 0.01 versus total number of faeces of the diseased group, one-way ANOVA, followed by Dunnett’s test.

*Nepeta ruderalis* crude extract showed antidiarrhoeal effect, when assessed in castor oil-induced diarrhoeal mice. Wet faeces plus the total number of faeces of each individual animal was counted to obtain the results. The animals in the negative control group were found to be diarrhoeal with 0% protection. While the animals that were administered 300 mg/kg dose of crude extract, exhibited 20% protection, but a significant effect was shown at 500 mg/kg with 60% protection. Whereas loperamide (10 mg/kg) and verapamil (50 mg/kg) also showed a significant effect with 100% protection against castor-oil induced diarrhoea (Table 1 and Figure 5).

**Table 1.** Percentage protection of crude extract of *N. ruderalis* (Nr.Cr) against castor oil (10 ml/kg)-induced diarrhoea in mice (*n* = 5).

Treatment (p.o.)	Mice with diarrhoea/total mice	Protection
Saline (10 ml/kg) + C. oil	5/5	0
Nr.Cr (300 mg/kg) + C. oil	4/5	20
Nr.Cr (500 mg/kg) + C. oil	2/5	60
Verapamil (50 mg/kg) + C. oil	0/5	100
Loperamide (10 mg/kg) + C. oil	0/5	100

## Discussion

Gastrointestinal motility is regulated by various physiological factors, like acetylcholine, histamine, serotonin, etc. associated with regulation of intracellular level of Ca^+2^ (Burks 1987). Screening was performed by using isolated tissue preparations as they are rationally used in such investigations to authenticate the underlying mechanism(s) of pharmacological effects, without any intrinsic (neural or hormonal) involvement (Mushtaq et al. 2015).

When screened for its possible spasmolytic effect, against its folkloric claims in gastric disorders (Quattrocchi 2012), crude extract of *N. ruderalis* showed a concentration-dependent spasmolytic effect in spontaneous contractions of isolated rabbit jejunum. An increase in intracellular Ca^+2^, associated with influx of Ca^+2^ through voltage-dependent calcium channels or discharge from the sarcoplasmic reticulum, causes the depolarization of smooth muscle (Berridge et al. 2003). The spontaneous movements of the intestine are mediated through repeated cycles of such depolarization (Ur-Rahaman et al. 2013).

It is well-established fact that the K^+^ at a concentration higher than 30 mM produces smooth muscle contraction by opening the voltage-dependent calcium channels that result in the influx of extracellular calcium. Any agent that relaxes the high K^+^-mediated contraction of the smooth muscle is considered to be a CCB (Bolton 1979). Carbachol (CCh): a muscarinic receptor agonist also exerts its contractile effect through activation of muscarinic receptors, associated with an increased level of intracellular Ca^+2^ (Qureshi et al. 2015). The crude extract completely inhibited CCh and high K^+^-mediated contractions, similar to verapamil, a known CCB (Michael & Hoffman 2011).

Nr.Cr also caused a significant (*p* < 0.05) rightward displacement of calcium CRCs at higher concentration (0.3 mg/mL), in isolated rabbit tracheal preparations, similar to that of verapamil (*p* < 0.01), indicating the presence of calcium channel-blocking activity behind the broncho-relaxant effect of *N. ruderalis*. As the CCBs have potential therapeutic effect in respiratory disorders like asthma and bronchitis (Ahmed 1992). So the calcium channel-blocking activity observed in the crude extract may provide the basis for the use of *N. ruderalis* as a bronchodilator in the traditional medicine.

In the small intestine, castor oil is converted into ricinoleic acid that stimulates peristaltic contractions and decreases intestinal absorption of electrolytes. The increased gastrointestinal motility is regulated by intracellular Ca ^+2^ that can cause diarrhoea (Mehmood et al. 2011a). So a CCB can be useful in hyperactive gut disorders by suppressing gastrointestinal motility (Khan et al. 2013). The presence of CCB like activity in crude extract indicates the effectiveness of *N. ruderalis* in diarrhoea and other hyperactive GI disorders (Janbaz et al. 2015). So the assumption was validated by *in vivo* experiments when a significant antidiarrhoeal protection (*p* < 0.01) was observed against castor oil-induced diarrhoeal mice, compared with the positive control drugs verapamil (*p* < 0.01) and loperamide (*p* < 0.01).

No acute toxicity symptoms were noted when *N. ruderalis* was given to healthy mice with increasing concentration of the crude extract up to 5 g/kg. The result may give the confidence about the safety of *N. ruderalis,* when used orally.

## Conclusions

The study validates the traditional uses of *the N. ruderalis* in hyperactive gastrointestinal and airway disorders, and the presence of calcium channel-blocking activity in the crude extracts can be perceived as the pharmacological basis behind its medicinal effects.

## References

[CIT0001] Ahmad VU, Bano S, Mohammad FV. 1985 Nepehinol – a new triterpene from *Nepeta hindostana*. Planta Med. 51:521–523. 17345277 10.1055/s-2007-969582

[CIT0002] Ahmad VU, Noorwala M, Mohammad FV, Shah MG, Parvez A. 1993 Nepehinal: a new triterpenoidal aldehyde from *Nepeta hindostana*. Planta Med. 59:366–368. 17235991 10.1055/s-2006-959703

[CIT0003] Ahmed T. 1992 Calcium antagonists: potential for asthma therapy. Choices Respir Manage. 22:41–43.

[CIT0004] Awan MH. 1960 Kitab ul Mafroodat. Lahore, Pakistan: Sheikh Ghulam Ali and Sons (pvt) Limited.

[CIT0005] Berridge MJ, Bootman MD, Roderick HL. 2003 Calcium signalling: dynamics, homeostasis and remodelling. Nat Rev Mol Cell Biol. 4:517–529. 12838335 10.1038/nrm1155

[CIT0006] Bolton TB. 1979 Mechanisms of action of transmitters and other substances on smooth muscle. Physiol Rev. 59:606–718. 37533 10.1152/physrev.1979.59.3.606

[CIT0007] Burks TF. 1987 Actions of drugs on gastrointestinal motility In: Johnson LR, editor. Physiology of the Gastrointestinal Tract. New York: Raven Press; p. 723–743.

[CIT0008] Chaudhary MA, Imran I, Bashir S, Mehmood MH, Rehman NU, Gilani AH. 2012 Evaluation of gut modulatory and bronchodilator activities of *Amaranthus spinosus* Linn. BMC Complement Altern Med. 12:166 23025418 10.1186/1472-6882-12-166 PMC3545920

[CIT0009] Clark JD, Baldwin RL, Bayne KA, Brown MJ, Gebhart GF, Gonder JC, Gwathmey JK, Keeling ME, Kohn DF, Robb JW, Smith OA. 1996 Guide for the care and use of laboratory animals. Washington, DC, USA: Institute of Laboratory Animal Resources, National Research Council.

[CIT0010] Janbaz KH, Hassan W, Mehmood MH, Gilani AH. 2015 Antidiarrheal, antispasmodic and bronchodilator activities of *Pistacia integerrima* are mediated through dual inhibition of muscarinic receptors and Ca^+2^ influx. Sci Tech Dev. 34:52–59.

[CIT0011] Janbaz KH, Latif MF, Saqib F, Imran I, Zia-Ul-Haq M, De Feo V. 2013 Pharmacological effects of *Lactuca serriola* L. in experimental model of gastrointestinal, respiratory, and vascular ailments. Evid Based Complement Alternat Med. 304394. 23662127 10.1155/2013/304394 PMC3638630

[CIT0012] Kew. 2014 An online resource for the world's plants [Internet]. c Board of Trustees of the Royal Botanic Gardens, Kew; [cited 2016 Jan 16]. Available from: http://wfo.kew.org/.

[CIT0013] Khan A, Gilani AH. 2009 Antispasmodic and bronchodilator activities of *Artemisia vulgaris* are mediated through dual blockade of muscarinic receptors and calcium influx. J Ethnopharmacol. 126:480–486. 19751814 10.1016/j.jep.2009.09.010

[CIT0014] Khan M, Shah AJ, Gilani AH. 2013 Antidiarrheal and antispasmodic activities of *Vitex negundo* are mediated through calcium channel blockade. Bangladesh J Pharmacol. 8:317–322.

[CIT0015] Khare CP. 2007 Indian medicinal plants: an illustrated dictionary. New Delhi, India: Springer.

[CIT0016] Koche D. 2010 Trace element analysis and vitamins from an Indian medicinal plant *Nepeta hindostana* (ROTH) Haines. Int J Pharm Pharm Sci. 3:53–54.

[CIT0017] Mehmood MH, Aziz N, Ghayur MN, Gilani AH. 2011a Pharmacological basis for the medicinal use of *Psyllium* husk (Ispaghula) in constipation and diarrhea. Dig Dis Sci. 56:1460–1471. 21082352 10.1007/s10620-010-1466-0

[CIT0018] Mehmood MH, Hasan SS, Gilani AH. 2011b The antidiarrheal and spasmolytic activities of *Phyllanthus emblica* are mediated through dual blockade of muscarinic receptors and Ca^2+^ channels. J Ethnopharmacol. 133:856–865. 21093572 10.1016/j.jep.2010.11.023

[CIT0029] Michael T, Hoffman BB. 2011 Treatment of myocardial ischemia and hypertension In: Brunton LL, Chabner BA, Knollmann BC, ed. Goodman and Gilman's The Pharmacological Basis of Therapeutics. China: McGraw-Hill Medical; p. 745–788.

[CIT0019] Mushtaq S, Chaudhry MA, Rahman HMA. 2015 Calcium channels blocked activity: providing the basis for medicinal use of *Abies pindrow* in diarrhea and bronchitis. Bangladesh J Pharmacol. 10:430–435.

[CIT0020] Nadkarni KM. 1996 Indian Materia Medica with Ayurvedic, Unani-tibbi, Siddha, Allopathic, Homeopathic, Naturopathic & Home Remedies, appendices & indexes. Bombay, India: Popular Prakashan.

[CIT0021] Naz SB, Chaudhary MA, Ul RMS. 2016 Dual receptor blocker mechanism arbitrates smooth muscle relaxant effect of *Polypodium vulgare* Linn. Bangladesh J Pharmacol. 11:414–420.

[CIT0022] Pandey AK, Mohan M, Singh P, Tripathi NN. 2015 Chemical composition, antioxidant and antimicrobial activities of the essential oil of *Nepeta hindostana* (Roth) Haines from India. Rec Nat Prod. 9:224–233.

[CIT0023] Quattrocchi U. 2012 CRC world dictionary of medicinal and poisonous plants: common names, scientific names, eponyms, synonyms, and etymology. Florida, USA: CRC Press.

[CIT0024] Qureshi HM, Omer OM, Ashraf M, Bukhsh A, Chaudhry MA, Imran MS. 2015 Evaluation of antihistaminic and anticholinergic activities of *Murraya koenigii* Linn. Pak Vet J. 35:242–244.

[CIT0025] Rehman NU, Khan AU, Alkharfy KM, Gilani AH. 2012 Pharmacological basis for the medicinal use of *Lepidium sativum* in airways disorders. Evid Based Complement Alternat Med. 596524. 10.1155/2012/596524 PMC3265128 22291849

[CIT0026] Ur-Rahaman MS, Chaudhary MA, Ahmad B, Alamgeer A. 2013 Rationalization of traditional uses of *Berberis lycium* in gastrointestinal disorders. Br J Med Med Res. 3:868–879.

[CIT0027] Van Rossum JM. 1963 Cumulative dose-response curves. II. Technique for the making of dose-response curves in isolated organs and the evaluation of drug parameters. Arch Int Pharmacodyn Ther. 143:299–330. 13996119

[CIT0028] Williamson EM, Okpako DT, Evans FJ. 1998 Selection, preparation and pharmacological evaluation of plant material. Chichester, West Sussex: John Wiley & Sons.

